# The ethics in sustainable AI: a scoping literature review on normativity in the academic discourse on the environmental sustainability of AI

**DOI:** 10.1007/s00146-026-02910-4

**Published:** 2026-02-28

**Authors:** Olya Kudina, Nynke van Uffelen, Lode Lauwaert, Wim Landuyt

**Affiliations:** 1 Delft University of Technology, Delft, Netherlands; 2 Optentia Research Unit, North-West University, Potchefstroom, South Africa; 3 KU Leuven, Leuven, Belgium

**Keywords:** Artificial intelligence, AI ethics, Sustainable AI, Sustainability of AI, Environmental impact of AI, Technofix

## Abstract

AI is developing rapidly, as are concerns about the environmental impact of its training and deployment. Studies about the environmental sustainability of AI have begun to emerge in the past five years, stressing the need for critical reflection on the discursive underbelly of this emerging scholarship. For example, how do authors frame the problem of the environmental impact of AI? Are there any ethical reflections accompanying their reporting, and if so, which ethical theories and principles guide normative considerations about the environmental impact of AI? In this study, we conduct a scoping literature review on (1) how authors refer to and frame the problem of the environmental impact of AI systems, (2) who is ascribed responsibility for mitigating said impact, (3) what mitigative measures are proposed, and (4) what normative commitments justify such prescriptive normative statements. Our findings indicate that most literature on the topic is concentrated in computer science, engineering, and natural sciences, and the humanities are mostly absent. This results in a dominance of technofix attitudes towards the problem, and a narrow and limited engagement with ethical principles and theories. As such, we argue for more interdisciplinary work on the environmental sustainability of AI, leading to more diverse solutions and more explicit and pluralistic ethical starting points, grounded, for example, in relational and more-than-human ethics. The findings of this review highlight gaps in the literature and opportunities for developers, social scientists, and AI ethicists for more effective and diverse responses to AI’s environmental impact.

## Introduction

While it is increasingly impossible to imagine our daily lives without Artificial Intelligence (AI), the numerous benefits of this technology are accompanied by significant environmental harms. Early studies report on the high energy costs of developing and using AI (Henderson et al. 2020; Lacoste et al. 2019; Strubell et al. 2020), amounting, according to some estimates (Shift project 2019), to larger CO2 emissions than those from the aviation industry. These environmental impacts grew more pronounced with the increased reliance on digital technologies during the pandemic, but it is the rapid adoption of Generative AI tools, such as ChatGPT, that brought these environmental costs into sharper focus. Not only do the CO2 emissions from AI-driven data center infrastructure tend to be grossly underestimated (O’Brien 2024), Generative AI also requires considerable water and energy usage. For instance, processing just 20–40 prompts in ChatGPT consumes about half a liter of water to cool the energy-intensive computations (Li et al. 2025), and writing a 100-word email requires as much energy as powering 14 LED lightbulbs for an hour (i.e., 0,14 kWh)(Verma and Tan 2024).

Regardless of this, the academic discourse so far has largely focused on the ideas and promises of Sustainable AI, or AI *for* sustainability, i.e., how AI can help to ameliorate negative climate change effects and help achieve the UN Sustainable Development Goals (Kamilaris and Prenafeta-Boldú, 2018; Miailhe et al. 2019; Nishant et al. 2020; Vinuesa et al. 2020; Zhang et al. 2018), to help track deforestation (Finer et al. 2018), to design energy-efficient buildings (Vattano 2014), to analyze soil conditions and optimize agricultural practices (Yubin et al. 2017), or to develop carbon capture technologies (Yan et al. 2021). The plea to explore the sustainability *of* AI, i.e., the environmental impacts of AI itself, is a recent one (Brevini 2020; Coeckelbergh 2021; Crawford 2021; Monserrate 2022; Van Wynsberghe 2021), urging to ground and materialize AI in the earth extraction practices, complex and environmentally tolling logistic chains, and crucially, in the energy- and water-demanding developmental and use practices.

Several authors have argued that the sustainability of AI is ethically relevant, as it brings about new ethical questions, dilemmas, and trade-offs (Bolte 2023; Bolte et al. 2022; Coeckelbergh 2021; Falk and Van Wynsberghe 2024; Robbins and van Wynsberghe 2022). In this paper, we build on these proposals to implement a new research agenda on the ethics of Sustainable AI. The term “sustainability” is known for interpretative flexibility, referring to the social, economic, environmental, and other (intertwined) dimensions (Giovannoni and Fabietti 2013; Kuhlman and Farrington 2010). Here, we focus on the environmental sustainability of AI, given the urgency of its ecological impacts. Our working definition refers to how the development and use of AI systems relate to minimizing environmental harm (e.g., resource depletion, emissions) and supporting long-term environmental well-being (European Commission 2021). We go beyond emphasizing the environmental impact of AI to examine its ethical meaning and implications for AI design and use.

The responsible development and use of AI are underpinned by systemic complexities (e.g., Kudina and Van De Poel 2024) and conceptual ambiguities on what both terms, “AI” and “sustainability,” mean (e.g., Samuel et al. 2022). Unraveling the discursive underbelly of research on AI sustainability is therefore crucial, as it clarifies the assumptions and directions shaping sustainability efforts. Accordingly, this study analyzes key argumentative strands in the literature on AI’s environmental impact: (1) how the impact is framed; (2) how responsibility is allocated; (3) which mitigation measures are proposed; and (4) the normative undertones accompanying these discussions.

Given the rapid development of the AI sector and its growing environmental footprint, understanding the normative assumptions in this literature is essential. We approach normativity as interdisciplinary and treat ethical and normative elements loosely and inductively, including evaluative language (e.g., “bad,” “better”), minimal references to obligation, and appeals to ethical theories and concepts, such as justice, responsibility, or transparency. Our aim is to elucidate the normative assumptions through which AI stakeholders justify practices and construct their narratives (Grin and Grunwald 2000; Grunwald 2014). To do this, Sect. 2 outlines the methodological foundation of the study. Section 3 presents findings on how AI’s environmental impact is framed, who is considered responsible, which solutions are proposed, and how normative references appear. Section 4 discusses these findings, with particular attention to technological fixes, dominant normative orientations, and the resulting conceptions of responsibility, identifying ethical and interdisciplinary opportunities. Section 5 presents brief conclusions.

## Methodology

In this paper, In this paper, we examine how ethical considerations are articulated within the growing scholarship on the sustainability of AI. While much existing research emphasizes the potential of AI to address ecological sustainability challenges (Kar et al. 2022; Nishant et al. 2020; Schoormann et al. 2023), we focus instead on the reverse perspective: how academic literature accounts for AI as a contributor to environmental harm and which normative assumptions underpin such accounts.

To this end, we employ a scoping literature review, a method within the family of systematic reviews (Arksey and O’Malley 2005; Colquhoun et al. 2014). Our aim is to identify normative insights in scholarly reporting on AI’s environmental impacts, scrutinize underlying assumptions, and reflect on the opportunities and limitations that emerge from these framings. In doing so, we also seek to better understand how the philosophy of technology engages with this interdisciplinary topic. Although empirical methods are increasingly used in philosophical research (Achterhuis 2001; Hämäläinen 2016; Pols 2023), systematic literature reviews remain relatively rare in the field (Wieczorek et al. 2023), due to their labor-intensive nature and the lack of large-scale reference corpora. Nevertheless, for an interdisciplinary domain such as philosophy of technology, such reviews can offer valuable cross-disciplinary insights and reveal trends and gaps that support further philosophical analysis.

A scoping review can be understood as a reconnaissance mission of a specified body of research. It is an exploratory method to grasp the nature, features, and size of specified scholarship, clarify its salient concepts and approaches, identify the knowledge gaps, and produce recommendations for further research (Arksey and O’Malley 2005; Colquhoun et al. 2014). Unlike systematic reviews, a scoping review does not usually start from a hypothesis that it aims to (dis)prove based on a large body of work, and does not require preregistration or a formal adherence to external guidelines^1^ (Munn et al. 2018). While flexible in design, scoping reviews remain systematic through the use of research protocols, exhaustive search strategies, and structured data extraction (Peters et al. 2015), ensuring transparency, reproducibility, and analytical reliability. Therefore, before detailing our search results, we will first describe each of our research steps, namely a search process, a relevance screening, and a clustering (see Table 1).

**Table 1:** An overview of the literature search, screening, and analysis process

Methodological steps	Description	Count papers	Authors involved	
Search process	1. Search protocol & criteria defined	Defined search terms combining synonyms for “environmental impact” and “AI” using PRE/1 logic	–	Author 1 (with co-authors)
	2. Scopus database searched	Search run on March 4, 2024	761	Author 1
	3. Language filtering	Non-English records excluded	742	Author 1
Relevance screening	4. Title and abstract screening	ASReview used (manual: Author 1; automated with plateau stopping: Author 2)	742	Authors 1 & 2
	5. Records excluded	Papers not focused on AI’s environmental impact removed	582	Authors 1 & 2
	6. Full-text articles initial assessment	Abstracts, intros, and conclusions reviewed	160	Authors 2 & 4
	7. Studies included for in-depth analysis	Final inclusion for qualitative coding and analysis	92	All authors
Analysis	8. Codebook creation & qualitative coding	Inductive coding based on normative content and environmental framing	–	Codebook – Author 2, coding and triangulation—all authors
	9. Analysis and synthesis	Guided by questions of framing, responsibility, solutions, and normativity	–	All authors

### Search process

We selected Scopus as the research database due to its broad interdisciplinary coverage and advanced search capabilities. To identify literature on the environmental impact of AI, we developed a search query combining terms related to “AI” and “environmental impact,” structured to reflect a causal relationship between them using the PRE/1 operator. After several iterations and joint author review, this process yielded the final search expression used in the systematic literature search:

(TITLE-ABS-KEY ( "environmental impact" OR "environmental effect" OR "ecological impact" OR "carbon footprint" OR sustainab* OR "water footprint" OR "GGE" OR "carbon emission*" OR water OR "climate change" OR "ecological footprint" OR "environmental consequence" OR "sustainability influence" OR "Environmental degradation" OR "Ecological consequence" OR "Environmental harm" OR "Climate change contribution") PRE/1 TITLE-ABS-KEY ( "artificial intelligence" OR "AI" OR "machine learning" OR "LLM" OR "GPT" OR "Intelligent Systems" OR "Natural Language Processing" OR "Deep Learning" OR "Text Generation" OR "Automated Speech Recognition" OR "Generative AI" OR "Neural Networks" OR "Reinforcement Learning" OR "Speech Recognition" OR "Computer Vision" OR "Predictive Analytics"))

Additionally, our search criteria focused on peer-reviewed papers (journal and conference), and book chapters, excluding monographs or pre-prints for feasibility. We ran the expression on March 4, 2024, identifying 761 documents. 99 papers were published between 1975 and 2018, and 679 papers dated from 2018 until March 4, 2024. Filtering to English yielded 742 papers for relevance screening. The majority of contributions were from Applied science (23% in Computer science, 15% in Engineering), next to Social science and humanities (8% in Social sciences, 10% in Environmental studies, 4% in Business and management), followed by Natural science (12%) and Formal science (8%). The remaining 19% of papers was not classified, although our analysis suggests it is predominantly of a technical nature.

### Relevance screening

An initial screening revealed that many papers, regardless of the search expression, focused on using AI for sustainability goals rather than examining AI’s own environmental impact. To support additional relevance screening, we used ASReview, a locally run open-source machine learning tool (Van De Schoot et al. 2021). ASReview allows reviewers to screen titles and abstracts against predefined criteria by labeling papers as relevant or irrelevant. Based on these decisions, the tool learns and prioritizes potentially relevant papers, pushing them to the top of the review stack. Screening can be stopped once a plateau of predominantly irrelevant papers is reached. To increase reliability and reduce interpretative bias, Authors 1 and 2 independently applied ASReview to the initial set of 742 papers. Their screenings showed an 81% overlap; all papers marked as relevant by at least one author were included, resulting in 160 articles for initial reading.

An initial reading of abstracts, introductions, and conclusions by Authors 2 and 4 further narrowed the corpus to 92 target articles (44 journal articles, 45 conference papers, 3 book chapters) for in-depth qualitative analysis, discarding papers that mentioned AI’s environmental impact only in passing or not at all. The temporal distribution mirrors the original set of 742 papers, remaining negligible before 2021 and increasing sharply thereafter (2011: 1; 2017: 1; 2020: 2; 2021: 9; 2022: 25; 2023: 46; 2024: 8). This pattern aligns with Bermejo and Juiz’s (2023) systematic review on AI in cloud/edge sustainability, who attribute this trend to the fact that “the topic is newborn and no work has yet been developed in this regard” (p. 52). Both stages of relevance screening were conducted in March-August 2024.

### Analysis

The following questions guided our analysis of the 92 target papers:

- How do the authors refer to the environmental impact of AI?
- Who are the actors that, according to the authors, should take responsibility for it?
- What are the proposed prescriptive solutions?
- How is normativity made apparent in this?

These questions were formulated based on screening the abstracts and the initial reading of the manuscripts. Since we were interested in the environmental impact of AI systems, reporting on this was almost always accompanied by mentioning the proposals on mitigating said impact and the ideas on who should do so. It is the reports and presuppositions on these subjects that hosted much of the normative narrative we were interested in. Therefore, we outlined the areas of framing, responsibility, solutions, and normativity as key research directions. Contributions were coded inductively, with codes and qualitative interpretations reviewed through co-author triangulation to ensure transparency and reliability. Qualitative analysis and synthesis were conducted in September-December 2024.

### Limitations

Before proceeding to the findings, we need to outline several limitations of this study. For instance, inevitably missing relevant search terms (Colquhoun et al. 2014) and potentially underrepresenting some fields (e.g., philosophy), relying on Scopus as the only database (Weinberg 2021). Topic-specific, in this quickly growing scholarship, it is difficult to disentangle works focusing on the environmental impact of AI systems from those studying Information and Communication Technology (ICT), digital, and cloud-based technologies in general. As environmental impact is always a complex property of large sociotechnical systems, by choosing to narrowly focus on AI systems (e.g., primarily algorithms and occasionally data centers), our study probably represents only the tip of the iceberg. Finally, this field is developing very rapidly. Running our search query before paper submission on January 6, 2025, yielded 1321 documents, and again, during final paper revisions on September 18, 2025, produced 2144 titles. So, from 2024 to 2025, the papers that fit the initial search criteria tripled. As our search and selection process shows, the majority would probably still be irrelevant to our study. Nonetheless, this significant increase indicates mounting attention to AI sustainability**.** In what follows, we examine the normative underbelly of this scholarship.

## Results

### How is the environmental impact of AI primarily referred to, if explicitly mentioned?

Our first question is how the problem of the environmental impact of AI is framed in the literature. This question is relevant since different framings can lead to different focuses in the research direction. When scholars approach the environmental impact of AI systems in terms of a cost problem, for example, more attention may be paid to efficiency, which may influence the proposed solutions.

First, we mapped what, according to the authors, constitutes the environmental impact of AI. Although all papers discuss high energy use and related CO2 and/or GHG emissions, our analysis reveals significant blind spots in the literature. Only 5 out of 92 papers mention water-related impacts, despite the considerable water footprint for cooling data centers (Li et al. 2024; Lannelongue and Inouye 2023; Bolte et al. 2022; Zhao et al. 2022; Robbins and van Wynsberghe 2022). Similarly, material resource challenges, including the extraction of critical/rare earth elements and electronic waste management, receive limited attention (N = 22) (e.g. Bolte et al. 2022; Ligozat et al. 2022). Most concerning is the near-absence of integrated environmental analysis, with merely 4 papers addressing both water and material resource impacts (e.g. Bolte et al. 2022; Zhao et al. 2022; Robbins and Van Wynsberghe 2022; Lannelongue and Inouye 2023), suggesting a fragmented approach that fails to capture the complex interdependencies of computing’s environmental footprint across multiple domains.

Second, we analyzed the aspects of the AI system that the contributions had in mind when mentioning the environmental impact of AI, i.e., the training and/or use of AI (see Table 2). According to Wu (2022), the training aspect encompasses all activities preceding actual implementation: collecting and processing large datasets, experimenting with different model architectures, and the actual training process, while the use phase involves the actual deployment of the trained model and inference (generating predictions) in practical environments. We classified articles as focusing on the training phase if the substantive analysis throughout the article concentrated on training-related environmental impacts, even if the authors acknowledged the impact of the use phase in introductory or contextual sections. For instance, Luccioni (2020) briefly mentions inference-related impacts but dedicates the focus almost exclusively to the environmental implications of model training; thus, we categorized this publication as such.

**Table 2:** An overview of the aspects of AI system the contributions refer to when mentioning the environmental impact of AI

Aspect of AI system vis-à-vis the environmental impact of AI	N = 92
Training (e.g., data gathering, experimentation, training)	38
Use (e.g., deployment, inference)	2
Training and use	49
Unclear	3

From our research results here, three findings stand out. First, a small majority of the studies (N = 49) adopt a holistic approach, focusing on the environmental impact of AI both in the training and deployment phases (e.g., Bannour et al. 2021; Borowiec et al. 2021; Nicodeme 2021; Walsh et al. 2021). Second, a substantial number of studies (N = 38) focus exclusively on the environmental impact of the training phase (e.g., Kindylidi and Cabral 2021; Luccioni et al. 2020; Puangpontip and Hewett 2023; P. D. Yoo et al. 2011). The prominence of this focus suggests that researchers identify the training phase as a critical point for intervention in sustainability efforts. Third, only 2 studies focus on AI’s deployment phase (Deng et al. 2023; Li et al. 2024). This underrepresentation is striking and suggests a blind spot in the field, given that the cumulative environmental impact of widespread AI applications over time could be substantial, especially for widely used models or systems.

Third, we analyzed how authors frame the environmental impact of AI, e.g. as a technical efficiency problem, a financial cost problem, an ethical problem, or a climate change problem (see Table 3). Nearly all contributions (N = 88) frame the environmental impact of AI as a problem for climate change, either exclusively or also implying other problems. The environmental influence is predominantly viewed as something that affects the climate, with greenhouse gas emissions and other resource-intensive properties of AI as the primary factors (N = 66). This majority indicates a dominant paradigm in how researchers conceptualize AI’s environmental footprint.

**Table 3:** Different framings of the problem of the environmental impact of AI

Framing per problem type (N = 92)	
An overall climate change concern (N = 88) that translates to…	A financial concern (N = 3)
	A technological concern (e.g. resource-intensive property of technology) (N = 66)
	An ethical concern (e.g. justice) (N = 19)
A financial concern (N = 4)	

19 studies frame the environmental impact of AI as a general ethical issue or specifically as a matter of justice (a predominant description in this account). These publications broaden the perspective by linking the environmental effects of AI to the normative questions about responsibility, justice, and moral obligations (e.g., Bolte et al. 2022; Budennyy et al. 2022; Draschner et al. 2022; Eluwole et al. 2022; Halsband 2022). They highlight aspects such as the unequal geographic distribution of AI systems’ environmental costs and benefits (with data centres often located in economically disadvantaged regions), the intergenerational injustice of resource depletion for short-term gains, and the tension between technological progress and planetary boundaries (details further in Tables 7 and 8). Overall, a normative approach to environmental issues in the AI context is underrepresented, with the field favoring an exclusively technical orientation (see Table 4 next).

**Table 4:** The proposed solutions to the problem of the environmental impact of AI

Proposed solutions	Specification	N = 150
Technological solutions (N = 65)	Software for energy efficiency/equity	47
	Renewable energy sources	6
	Improving hardware (servers, computers, data centers)	12
Procedural/institutional solutions (N = 37)	(Ethical) reflection, participation and/or deliberation	22
	Policies, regulations, guidelines, standards, certifications, labels	15
Transparency tools/methods (N = 35)	Proposing a tool/method for tracking environmental impact	15
	Developers need to report on the environmental impact	10
	Review of methods/tools to calculate environmental impact	5
	There is a need for methods/tools to calculate environmental impact	3
	Transparency is necessary for developers to make more sustainable choices	2
Other	Conceptual recommendations (e.g. to reconceptualize sustainability or AI)	6
	A choice not to develop an AI system	3

Economic and financial perspectives are even more underrepresented, as only 7 studies primarily conceptualize this impact as a financial issue, of which 4 exclusively focus on cost aspects (Nixon et al. 2023; Spring and Shrivastava 2017; Bai et al. 2024; Agliari et al. 2024) and 3 combine environmental and cost concerns (Nicodeme 2021; Rafat et al. 2023; Wang 2023). Research within this category addresses topics such as the economic costs of energy consumption, the financial implications of regulatory measures, the cost-effectiveness of various sustainability strategies, and the relationship between energy costs and computational budgets for AI development. The limited explicit attention to economic dimensions is remarkable, given that financial considerations often play a decisive role in decision-making regarding technological implementations within organizations. This may indicate a gap between academic research and practice-oriented decision-making, where economic factors might play a larger role than in scientific conceptualizations.

### Proposed solutions

Normativity also features in the solutions that the authors propose. The proposed solutions, in this sense, are prescriptive normative statements, stating what ‘should’ or ‘ought to be done.’ An overview of the proposed solutions can be found in Table 4—note that authors may propose more than one solution in one paper, hence a larger number of solutions (N = 150) compared to the total number of papers analyzed (N = 92).

The first category of proposed solutions is *technological* in nature. Here, software optimization solutions make up the largest category of prescribed ways of addressing the environmental impact of AI systems (N = 47). This category includes papers that argue for the need for more energy-efficient software to reduce the environmental impact of AI (e.g., Dokic et al. 2023; Ray and Das 2023; Remedios et al. 2023); recommendations for using specific tools or strategies that increase energy efficiency (e.g., Selvan et al. 2022); and proposals for energy-efficient software, approaches, and models developed by the authors (e.g., Yoo and colleagues [2011] develop an energy-efficient framework for large-scale data modelling and classification). A second type of proposed technological solutions pertains to improving the hardware on which AI relies, such as the servers and computers in data centers, which can be produced in more sustainable ways (N = 13)(e.g., Arquilla and Paracolli 2023; Wang 2023; Islam et al. 2023). Lastly, 6 contributions argue for the importance of using renewable energy sources for data centers to reduce the carbon emissions generated by AI (Alloghani 2024a; An et al. 2023; Draschner et al. 2022; Eluwole et al. 2022; Güler and Yener 2021; Ray and Das 2023).

The second category of solutions pertains to the *processes and institutions* around AI development. On the one hand, 22 contributions argue for the need for more *reflection and/or deliberation* on the environmental impact of AI. Some authors plead for more conversation, reflection, and collaboration on environment-related biases in thinking (Myllylä, 2022) and on the environmental impact of AI amongst developers (e.g., Dokic et al. 2023; Lammert et al. 2023; Lannelongue and Inouye 2023; Liao et al. 2023; Remedios et al. 2023; Rohde et al. 2024; Weber et al. 2023), AI artists (Deng et al. 2023), within the AI community (Eilam et al. 2023; Yoo et al. 2023), amongst management (Mill et al. 2022), or within society at large (Borowiec et al. 2021). Two papers argue for global stakeholder engagement and participation (Banipal et al. 2023; Vinuesa et al. 2020). Another 7 contributions explicitly stress the need for ethical reflection, deliberation, dialogue, and assessment of AI given its environmental impact and in relation to its potential benefits (Bolte et al. 2022; Halsband 2022; Moyano-Fernández and Rueda 2023; Ray and Das 2023; Richie 2022; Robbins and van Wynsberghe 2022; van Wynsberghe et al. 2022). On the other hand, 15 articles explicitly call for more *policies, regulations, guidelines, standards, certifications, codes of ethics, or labels* for AI, either to guide AI development (Alloghani 2024b, 2024a; Banipal et al. 2023; Bermejo and Juiz 2023; Cowls et al. 2023; König et al. 2022; Nordgren 2023; Perucica and Andjelkovic 2022; Prakash et al. 2023; Ray and Das 2023), to enforce that developers report on the environmental impact of their models (Kindylidi and Cabral 2021; Vinuesa et al. 2020), or for both reporting and (self-)assessment within businesses (Cowls et al. 2023; Hacker 2023; Rohde et al. 2024).

In the third category, 32 contributions consider more transparency about the environmental impact of AI as a solution to the problem. Three contributions stress the need for tools or methods for tracking or measuring the environmental impact of AI (Bai et al. 2024; Bratan et al. 2024; Dokic et al. 2023); 15 contributions propose such tools (e.g., Liao et al. 2023; Luccioni et al. 2020; Pirnat et al. 2022); 10 contributions argue that developers need to report on the environmental impact of their models, for example, because transparency would allow developers to make more sustainable choices (e.g., Banipal et al. 2023; Debus et al. 2023; Draschner et al. 2022; Ferro et al. 2023; Gaur et al. 2023); and 5 contributions offer a review of existing methods and tools (Bannour et al. 2021; Bouza et al. 2023; Kunkel et al. 2023; Rajkumar 2022; Selvan et al. 2022).

Of the 92 papers, 6 papers attempt to reconceptualise a concept as a solution, such as sustainability (Bolte et al. 2022; Halsband 2022; Hessenthaler et al. 2022; van Wynsberghe et al. 2022), or AI itself, as an infrastructure (Robbins and van Wynsberghe 2022) or as part of a larger ecological and sociotechnical system (Bolte 2023). Notably, only 3 papers explicitly mention choosing not to develop or deploy an AI system as a potential solution (Bolte et al. 2022; Richie 2022; Robbins and van Wynsberghe 2022). For example, Richie (2022) considers the environmental impact of AI in healthcare and asks whether the benefits of AI always outweigh its negative impact on the environment. She questions and critiques “the assumption that all available health care technologies are medically necessary and therefore carbon emissions are morally irrelevant” (Richie 2022, p. 548). Moreover, Bolte and colleagues (2022) argue for an “ethics of desirability” approach to the problem, which implies questioning the necessity and justification of AI systems from the beginning instead of aiming at the design of particular AI systems and assuming the inevitability of AI development in general (i.e., technological determinism). In a similar vein, Robbins and van Wynsberghe (2022) argue that we must ask “inconvenient questions regarding these environmental costs before becoming locked into this new AI infrastructure” (p. 31). As such, the authors suggest that “The question before any AI model is created should be: is this worth the environmental cost that we will be locked into for decades? The answer will often be no” (Robbins and van Wynsberghe 2022, p. 39).

Based on the prescriptive statements in the contributions and their framings, we coded the papers as oriented towards optimism about AI’s capacity to sufficiently mitigate its environmental impacts or pessimism towards AI’s capacity to become sufficiently sustainable (Table 5). Most contributions seem to feature an optimistic stance, as their authors seem to assume that AI can be more sustainable or equitable, provided their recommended solutions are followed (N = 82). A total of 5 papers, however, do not make such claims and reflect a more pessimistic intuition. This includes two conceptual-philosophical contributions (Richie 2022; Robbins and van Wynsberghe 2022), but also contributions that stress the urgency of engineering and policy solutions to mitigate worst-case scenarios (Hessenthaler et al. 2022; König et al. 2022; Perifanis et al. 2023). The remaining 5 contributions did not feature the optimism/pessimism orientation, which we classified as “neutral.”

**Table 5:** An overview of attitudes towards AI systems

Attitude towards AI systems	N = 92
Optimistic—AI can be more sustainable	81
Optimistic—AI can be more equitable	1
Pessimistic	5
Neutral	5

### Who should take responsibility?

Another dominant category of analysis was “responsibility,” descriptively, as the one that frequently and even several times appeared next to discussing the environmental impact of AI and solutions to it, and normatively, as the actors who, according to the contributors, should take responsibility for mitigating the environmental impact of AI systems (N = 127 of responsibility ascription in 92 analyzed papers). The contributors of the papers ascribed responsibility for AI’s environmental footprint to multiple possible stakeholders: developers, researchers (both in engineering and AI ethics), policymakers, consumers, and many others. The results (Table 6) show a clear tendency to assign responsibility primarily to developers, with 73 contributions sharing this view. Engineering researchers follow at a distance as second, with 22 papers assigning responsibility to them (Budennyy et al. 2022), while policymakers are identified by 17 papers (Perucica and Andjelkovic 2022). Notably, few contributions assign responsibility to the users of AI (N = 6) (Bannour et al. 2021; Deng et al. 2023; Frey et al. 2022; Kindylidi and Cabral 2021; Rohde et al. 2024; Zhao et al. 2023) or to researchers in AI ethics (N = 4) (Banipal et al. 2023; Ray and Das 2023; Richie 2022; Robbins and van Wynsberghe 2022). This distribution suggests an upstream approach to responsibility, with emphasis on the source of the technology rather than the end users or regulatory bodies.

**Table 6:** The actors deemed responsible for addressing the problem of the environmental impact of AI

Who should take responsibility	N = 127
Developers	73
Engineering researchers	22
Policymakers	17
Users	6
AI ethics researchers	4
Multiple disciplines	3
Unclear	2

### Ethical puzzles and ways of addressing these

Finally, we reviewed (a) whether authors note explicit ethical dilemmas, questions, and issues in relation to the environmental impact of AI, and (b) whether and which normative commitments and principles are adopted in the face of these ethical issues and questions.

First (a), we searched for explicit mentions of ethical problems, issues, dilemmas, or trade-offs in relation to the environmental impact of AI (for an overview, see Table 7—note that authors frequently mentioned more than one ethical problem or question). We found that 57 contributions do not report any specific ethical issues, other than departing from the environmental impact of AI as a (financial, ethical, or technical) problem. As such, these contributions can be taken as addressing a minimal ethical concern, namely the problem of the environmental impact as contributing to climate change, which the authors deem as ethically undesirable.

**Table 7:** The ethical puzzles mentioned in the selected contributions

Ethical category	Description of an ethical problem, issue, dilemma, or trade-off	N = 98
Minimal ethical concern (N = 57)	The environmental impact of AI as a problem	57
Trade-offs (N = 25)	Costs/environmental impact—benefits trade-off	8
	Energy/environment—accuracy trade-off	6
	Sustainability of AI—AI for sustainability trade-off	4
	Sustainability—privacy trade-off	2
	Environmental impact—fairness trade-off	1
	Collective—individual benefits trade-off	1
	CO2—competitiveness trade-off	1
	Energy—training speed/security trade-off	1
	Improving health with AI—damaging health with the environmental impact of AI trade-off	1
Injustices (N = 4)	Environmental injustice (responsibility gap)	1
	Intergenerational justice	1
	Local/regional inequity	2
Other ethical questions/issues (N = 12)	Rebound effect / Jevons paradox	3
	Is AI really necessary/justified?	2
	What future do we want?	3
	What socio-political (power) structures and paradigms does AI reproduce?	1
	Responsibility gap for mitigation/adaptation between countries	1
	Proportionality of AI regulation?	1
	Research fairness	1

A total of 24 contributions explicitly articulate a trade-off between the negative and undesirable environmental impact of AI on the one hand and the benefits that AI might have on the other hand. When explicitly mentioned, the benefits of AI include the accuracy of the model, sustainability, privacy, fairness, competitiveness, training speed, security, and health. Interestingly, 4 papers mention a trade-off between the sustainability of AI and AI for sustainability, pointing out that AI models for sustainability also have an environmental impact (Borowiec et al. 2021; Cowls et al. 2023; Ligozat et al. 2022; Nordgren 2023). A particularly interesting trade-off was discussed by Lannelongue and Inouye (2023): “how to balance trying to cure diseases [with the help of AI] with the health impacts of climate change, partly fueled by large data centers?” Also worth noting is the paper by Hessenthaler and colleagues (2022), which points out that improving the environmental impact of AI may also have unfair consequences, which stresses the need to carefully scrutinize efforts for sustainable AI development for potential unfair consequences. Moreover, 4 contributions articulate injustices in relation to the environmental impact of AI (Eluwole et al. 2022; Halsband 2022; Li et al. 2024; Taddeo et al. 2021). Taddeo and colleagues argue that the environmental impact of AI is frequently “felt more severely by marginalized communities and in poorer regions, less responsible for climate change in the first place” (2021, p. 778). Halsband (2022) discusses how AI’s environmental impact creates injustices for future generations. Finally, Eluwole and colleagues (2022) point out local injustices that result from data centers, such as “heat, noise, and landscape interference/impact,” while Li and colleagues (2024) further elaborate on local and regional environmental inequities that result from AI infrastructure.

Lastly, eight other ethical issues were mentioned in relation to the topic. For example, three papers mention rebound effects, or Jevons paradox, pointing out that increased energy efficiency may result in an increase in the use of AI (Bratan et al. 2024; Ligozat et al. 2022; Selvan et al. 2022). Several papers ask philosophical-ethical issues, such as whether AI is necessary and justified in a specific context (Bolte et al. 2022; Richie 2022), what future we want (Bolte 2023; Robbins and van Wynsberghe 2022; van Wynsberghe et al. 2022), and what socio-political power structures and paradigms AI reproduces (Bolte et al. 2022). Lastly, Nordgren and colleagues (2023) argue that high-income countries have more responsibilities in taking mitigation and adaptation measures than low-income countries, thus pointing out a global responsibility gap as an ethical issue.

Second (b), we scrutinized whether and which normative commitments and principles authors adopt, facing the ethical puzzles that arise from AI’s environmental impact (as seen in Table 7). Some normative commitments and principles are implicit in the texts. For example, from the solutions as presented in Sect. 3.2, we deduce that all papers reveal an implicit commitment to making AI more sustainable, in other words, to a value of sustainability. Moreover, the technological solutions refer to an implicit normative commitment to AI as a valuable technology; authors who propose more reflection, participation, and deliberation subscribe to intellectual and democratic values; and authors proposing or stressing the need for more transparency and reporting consider transparency as either intrinsically or instrumentally important. In what follows, we present and discuss the *explicit* normative commitments, concepts, principles, and theories mentioned by the authors in the dataset (see Table 8), beyond commitments to different types of solutions.

**Table 8:** The explicit normative principles and concepts mentioned in the contributions in the dataset

Normative cluster	Normative principles and concepts	N = 17
Justice (N = 11)	Fairness (e.g., reducing stereotypical bias)	1
	Research fairness	1
	Fairness (vis-à-vis transparency and security)	1
	Justice (e.g. health, resource conservation, no-harm)	1
	Social justice (e.g., principles of transparency, interpretability, and fairness), minimizing harm, human-centric engineering	1
	Intergenerational relations/justice	1
	Sustainability as global ecological, social distributional, and inter- and intragenerational justice; just distribution of opportunities and harms; compliance with planetary boundaries	1
	Fairness and equity, recognition, and utility (e.g. vis-à-vis environmental ethics approaches, transparency, efficiency)	1
	Intra- and intergenerational justice (e.g. sustainability, avoiding lock-in, environment as a starting point)	1
	Environmental inequity, social-ecological justice, fair balance of regional environmental impact (e.g., minimize AI’s highest environmental cost across all the regions)	1
	Equity, against utilitarianism	1
Utilitarianism (N = 3)	Maximizing gain, minimizing impact	1
	Increase long-term benefits and quality of life for humans and environment	1
	Maximizing performance metrics (e.g., recall, accuracy) and minimizing environmental impact	1
Other (N = 3)	Normative demands of sustainability	1
	Ethics of desirability	1
	Preventing harm, vulnerable relationships, and information asymmetries	1

In our dataset, 3 contributions explicitly adhere to maximizing ‘positives’ and minimizing ‘negatives,’ or harm, which can be understood as a utilitarian normative commitment (Escarda Fernández et al. 2023; Ligozat et al. 2022; Myllylä, 2022). For example, Myllylä et al. argue that designers ought to “increase the long-term positive benefit and quality of lives for humans and environments, at the individual, group, local, and global levels” (2022). These contributions contrast with 11 articles that foreground justice, equity, or fairness in relation to sustainability. For example, Vinuesa et al. explicitly critique utilitarian approaches because “positive societal welfare implications [of AI] may not always benefit each individual separately” (Vinuesa et al. 2020, p. 6). As such, utilitarian approaches do not suffice for dealing with trade-offs between the distribution of burdens and benefits between individuals and collectives. Instead, “To deal with the ethical dilemmas raised above, it is important that all applications provide openness about the choices and decisions made during design, development, and use […]. It is therefore important to adopt decentralized AI approaches for a more equitable development of AI” (Vinuesa et al. 2020, p. 7).

Of the 11 contributions that explicitly mention justice (that we cluster with fairness and equity), 3 focus on distributive justice (Richie 2022; Rohde et al. 2024; Li et al. 2024), which concerns the just distribution of goods, services, burdens, and benefits. Richie focuses on ‘justice’ as a principle in bioethics, which revolves around distributive justice as “mitigating gaping disparities in health care delivery” (Richie 2022, p. 551). Rohde and colleagues consider distributive justice as a part of sustainability, and consider “a just distribution of resources, opportunities and harms” (2024, p. 5). Li and colleagues are concerned with environmental inequity, referring to “the fact that AI’s environmental footprint can be disproportionately higher in certain regions than others,” and propose equity-aware geographical load balancing to “minimize AI’s highest environmental cost across all regions” (Li et al. 2024, p. 1). Other contributions remain rather general and do not define justice or fairness further than mentioning general principles. For example, Lammert and colleagues (2023) argue that social justice includes “principles such as transparency, interpretability, and fairness”; Remedios and colleagues (2023) argue that sustainability includes “aspects like fairness, transparency, and inclusivity”; and Hessenthaler and colleagues (2022) consider fairness only in relation to stereotypical bias in AI. Two contributions explicitly counter anthropocentric approaches to justice, taking (ethics or justice for) the environment as a starting point (Moyano-Fernández and Rueda 2023; Robbins and van Wynsberghe 2022). In addition, two articles stress the need for considering intergenerational justice in relation to the environmental impact of AI (Halsband 2022; Robbins and van Wynsberghe 2022). Lastly, one article thematizes fairness vis-à-vis research diversity, arguing that “The need for high computing power brings higher carbon emission and undermines research fairness by preventing small or medium-sized research institutions and companies with limited funding in participating in research” (Zhou et al. 2023). Few contributions that mention justice, fairness, or equity explicitly engage with theories of justice or authors within political philosophy or theory.

In the ‘other’ category, Alloghani (2024b) mentions “preventing harm, vulnerable relationships, and groups in which information asymmetries may ensue between a business and the consumers” as ethical principles. Moreover, Bolte et al. (2022) conceptualizes an *ethics of desirability*, which implies moving away from an *ethics of carefulness* or a ‘checklist ethics’ that assumes AI is an inevitable technological development, towards questioning the technological paradigm altogether and bringing back ethical and social-political choices in relation to AI development and use. Lastly, in a conference paper, Bolte (2023) aims to explore the normative demands of sustainability.

## Discussion

The findings of this scoping review evoke reflection along several dimensions regarding the main research focus of this paper, i.e., uncovering the normative side of scholarship on the environmental sustainability of AI. Below, we delineate several specific lines of discussion, namely regarding the conceptual ambiguity and language pragmatics, the disciplinary scope, the domination of technofix solutions, the specific ethical dilemmas and issues, and the normative dominance of principle-based and anthropocentric approaches.

### Conceptual ambiguity and language pragmatics

Although not an explicit search category, our analysis consistently encountered conceptual ambiguity surrounding the meanings of both “AI” and “sustainability,” as well as the language through which the environmental impact of AI was framed. These language pragmatics often carried implicit normative expectations and shaped how AI and its impacts were understood.

*Regarding the term “AI,”* the literature showed a relatively shared orientation toward concrete technical implementations rather than treating AI as an abstract concept. Across the 92 papers analyzed, we identified approximately 100 distinct technical implementations, tools, or system-level interventions aimed at mitigating AI’s environmental impact, indicating that several papers proposed multiple technological solutions. This reflects a strong emphasis on applied AI.

Relatedly, our findings reveal an overwhelming dominance of technological framings of AI, with limited attention to its social, institutional, or environmental embeddedness. Environmental concerns were largely treated instrumentally, in terms of resource depletion or replenishment. Only a small subset of papers, predominantly philosophical contributions, explicitly framed AI as a sociotechnical system operating across social, technological, institutional, and environmental dimensions (e.g. Bolte 2023; Ray and Das 2023; van Wynsberghe et al. 2022; Taddeo et al. 2021). This aligns with long-standing calls in philosophy of technology and STS to conceptualize AI as a complex sociotechnical system whose ethical opportunities and challenges emerge from interactions between system components (e.g. Johnson and Verdicchio 2017; Brevini 2020; Van Wynsberghe 2021; Crawford 2021; Rakova and Dobbe 2023; Kudina and Van De Poel 2024). However, our review highlights a persistent disconnect between this systemic framing and how AI is treated in most environmental impact studies, where it is still approached in narrowly technological terms. This gap warrants further interdisciplinary investigation, particularly given AI’s broad adoption and the expectations surrounding it.

Conceptual ambiguity was even more pronounced *regarding the term “sustainability.”* The analyzed papers rarely shared a common conceptual framework, complicating efforts to identify a coherent body of work on AI’s environmental impact. Despite appeals to use “Sustainable AI” as an umbrella term encompassing both AI for sustainability and the sustainability of AI (Van Wynsberghe 2021), the literature overwhelmingly emphasized the former. Consequently, references to “Sustainable AI” often proved counterproductive for identifying studies focused on AI’s environmental footprint. To distinguish these framings, we used the terms “sustainability of AI” or “AI sustainability” to underscore the effects of AI on the ecological environment, with downstream implications for humans and non-human animals.

When environmental impacts were discussed, they primarily concerned resource-intensive AI training and use, with a strong focus on electricity consumption; water use and hardware production were rarely addressed. Notably, only one paper (Ligozat et al. 2022) explicitly engaged with sustainability assessment standards defined by the International Organization for Standardization (ISO 14040 and 14,044). Greater engagement with such standards could help clarify concepts, align expectations, and distribute responsibilities more coherently within the highly interdisciplinary field of AI sustainability.

### Disciplinary scope: where are the humanities?

Although AI development in general, and the sustainability of AI in particular, is increasingly interdisciplinary, scholarship on AI’s environmental impact remains largely concentrated in computer science, engineering, and the natural and formal sciences (see Sect. 2). The relatively few contributions from philosophy and the social sciences are mostly position papers (Bolte et al. 2022; Coeckelbergh 2023; Van Wynsberghe 2021), programmatic reflections, or broad guidance frameworks (Cowls et al. 2023; Taddeo et al. 2021). Notably, among the four papers that explicitly address injustice as an ethical issue (Sect. 3.4), two are authored solely by computer scientists (Eluwole et al. 2022; Li et al. 2024). This disciplinary distribution is not unexpected given the relatively recent recognition of AI’s environmental impacts. Although our search yielded 22 philosophy and social science papers linking AI and sustainability, most focused on other sustainability dimensions and were therefore excluded.

Disciplinary scope is closely connected to the types of solutions proposed and the actors deemed responsible. The dominance of technical disciplines corresponds with a prevalence of technological fixes and an emphasis on responsibility at the level of developers in research and industry, with limited attention to policy, governance, or ethical analysis. Yet sustainability challenges related to AI increasingly take the form of complex sociotechnical problems that cannot be attributed to single actors (e.g., Crawford 2021; Kudina and Van De Poel 2024; Robbins and van Wynsberghe 2022), requiring coordinated interdisciplinary responses.

While interdisciplinary collaboration in AI development is widely acknowledged as difficult, efforts to address this challenge are growing (e.g., Kusters et al. 2020; Zeller and Dwyer 2022; Bisconti et al. 2023; Schleiss et al. 2025). Our findings underscore the need for deeper engagement from the social sciences and humanities in research on AI sustainability. Such work could include case studies, attention to local and political contexts, and analysis of structural conditions, thereby broadening existing technical research. It could help to recognize the presence and normative importance of different actors and salient factors (e.g., more-than-human actors, see Sect. 4.5 further), sensitivity to different value frameworks and local contexts. Combined with technical problem-solving, an interdisciplinary approach can thus render sustainability solutions more specific, inclusive, and actionable.

### Technofix attitudes

Many of the reviewed articles follow a similar structure: AI is framed as an environmental problem primarily due to CO₂ emissions associated with the energy required for its development and use; consequently, developers need to devise technological solutions to make AI more sustainable. A substantial portion of the literature identifies this as a transparency problem: if environmental costs are properly tracked and measured, the issue is assumed to be mitigated. This framing guides much of the scholarship toward developing technical solutions, tracking matrices, and reporting tools that quantify electricity use and translate it into greenhouse gas emissions. Moreover, environmental impacts are often treated as siloed, pertaining to a single component of the AI system, often an algorithm or computational unit, rendering the proposed solutions similarly isolated and predominantly technical.

The dominance of certain disciplines and their perspectives shaping the field partly explains this orientation. While we do not deny the potential value of the proposed technical solutions, nor assess their technical effectiveness, we highlight the risks of an overarching technological solutionism that characterizes much of the sustainability-of-AI literature. Following Sætra and Selinger, we define a technofix as a “technological remedy for a social problem that only involves minimal use of political power or economic incentives to change an individual’s behaviour and does not require changing social norms” (2024, p. 7).^2^ Philosophy of technology scholarship has extensively documented the broader implications of such approaches, for example, leading to “the economisation and depoliticisation of planetary environmental issues” (Dillet & Hatzisavvidou 2022), “climatic instability” (Veraart et al. 2023), and “absolv[ing] actors from taking more difficult steps toward systemic solutions” (Wagner and Zizzamia 2022). Primarily, a technofix attitude narrows the ethical space of inquiry, sidelining fundamental questions (e.g., Bolte et al. 2022; Richie 2022; Robbins and van Wynsberghe 2022): Is AI application necessary or valuable, and for whom? Are its environmental costs justified? Does deploying AI for sustainability risk “fighting fire with fire”? And what kind of future do such developments promote?

An important feature of the technofix logic is its treatment of responsibility. Although our analysis of responsibility was initially descriptive (see Sect. 3.3), it also has normative implications. Technological fixes often presume a clearly identifiable problem owner whose task is to resolve the issue. However, the environmental impact of AI is better understood as “a problem of many hands” (Van de Poel et al. 2012), arising from the interaction of multiple actors, infrastructures, and institutions. Another problem with the responsibility focus is that it overshadows alternative ethical readings of the problem. A different ethical lens, for instance, that of virtue ethics, could focus on the moral character of the actors involved (instead of or next to their responsibility and liability) and prospectively guide the next generation of AI developers (Harris 2008; Bergen and Robaey 2022). Broadening ethical analysis beyond responsibility can therefore enrich understanding of AI sustainability.

In line with our earlier discussion of disciplinary scope, and echoing conclusions across the literature (e.g., Nicodeme 2021; Perucica and Andjelkovic 2022; Ray and Das 2023; Rohde et al. 2024; van Wynsberghe et al. 2022), we argue that a focus on technological solutions alone is insufficient to address the social, ethical, political, and environmental dimensions of AI sustainability. While interdisciplinary and systemic inquiry may be impractical or unfeasible for individual researchers, even acknowledging the AI system complexity and the multidimensional nature of AI sustainability as grounding points and promoting these visions in disciplinary arenas can go a long way. It could motivate individual researchers to seek out approaches not reflected in their own work but complementing it, deepening their contributions.

Such recognition also supports collective research models, including interdisciplinary consortia, that reflect the multifaceted nature of AI’s environmental impact. Funding bodies increasingly promote such collaboration to address complex problems and enhance research impact. The environmental sustainability of AI similarly calls for strategic division of labor combined with coordination, ensuring that disciplinary contributions form a coherent and cumulative body of knowledge. Existing collaborative efforts in AI development (e.g., Kusters et al. 2020; Zeller and Dwyer 2022; Bisconti et al. 2023; Schleiss et al. 2025) provide a foundation for advancing comparable initiatives focused specifically on AI sustainability.

### Ethical dilemmas that need engagement and reflexivity

Regardless of the general lack of explicit ethical reflection, there are very interesting ethical dilemmas and issues described in the literature, such as trade-offs between the environmental impact of AI and the benefits of AI systems, including AI for sustainability, healthcare, privacy, accuracy, and efficiency. Addressing these dilemmas is often relegated to the need for a technofix to reduce the energy consumption of AI, or to a need for additional developers’ and business reflexivity. Although a welcome call, often, this seems to place a considerable toll on AI designers (Myllylä 2022).

As such, we see an opportunity for philosophers and AI ethicists to engage more deeply with concrete ethical dilemmas and trade-offs that arise in concrete AI applications. Most conceptual-philosophical papers in the dataset give general indications or directions (e.g., Bolte et al. 2022; Coeckelbergh 2023; Van Wynsberghe 2021). We see opportunities to further develop such proposals, potentially within collaborations between developers and ethicists. One pitfall of this proposal may be that a sole focus on concrete AI applications overlooks structural issues that the system as a whole brings about. As such, we do not advocate a sole focus on applied AI research on the environmental sustainability of AI, but rather for a larger and more diverse ethical research landscape on the topic, as we discussed in Sect. 4.2.

### Normative dominance of principle-based and anthropocentric approaches: where is more-than-human ethics?

Our findings show that explicit ethical referencing remains rare in scholarship addressing the environmental impact of AI, even when authors directly discuss its negative or undesirable effects. This can partly be explained by the interdisciplinary composition and relatively nascent state of the field. In itself, the absence of ethical standardization is not necessarily problematic. For instance, the field of Responsible AI is currently drowning in AI ethics, predominantly understood as rules, guidelines, and and frameworks that often talk past one another and prove difficult to implement (e.g., Bolte et al. 2022; Hagendorff 2020; Mittelstadt 2019; Segun 2021). From this perspective, limited ethical proceduralism in the AI sustainability scholarship may appear advantageous. But procedures are not all there is to ethics.

The absence of explicit ethical articulation poses significant risks. Normative commitments do not disappear when left unstated. Instead, they continue to implicitly shape problem framings and proposed solutions, complicating the identification and mitigation of inevitable biases (Myllylä, 2022; Van Uffelen et al. 2024). For example, the assumption that AI’s environmental impact is justified insofar as it benefits “us.”

Across the literature reviewed, ethical reasoning is predominantly anthropocentric. Environmental sustainability is framed primarily as instrumental to human well-being, as a resource. Moreover, the solutionist logic identified earlier, focusing on energy or algorithmic optimization, fails to capture the systemic interdependencies that characterize AI development and deployment. This reinforces a perception of technological primacy over social and environmental dimensions and aligns with backward-looking models of responsibility focused on identifying and allocating harm (Young 2003).

Only 17 of the 92 analyzed contributions explicitly referenced ethical principles or commitments (Alloghani 2024b; Lammert et al. 2023; Remedios et al. 2023; Richie 2022), with distributive and intergenerational justice being the most frequently invoked lens (e.g. Halsband 2022; Li et al. 2024; Richie 2022; Rohde et al. 2024). This mirrors broader trends in ethics applied to complex sociotechnical problems, where principle-based approaches dominate (Floridi et al. 2018; Von Schomberg 2011; Boenink and Kudina 2020). Justice-based approaches are also increasingly influential in assessing sociotechnical systems, emphasizing their distributive, procedural, restorative, and recognition-based dimensions (Costanza-Chock 2018; Van Uffelen et al. 2024).

Despite their strengths to procedurally streamline and normatively adjudicate developmental practices and resulting technologies, principle-based approaches exhibit notable limitations, related to a focus on isolated system components (Weber et al. 2023; Wood 2023; Rakova and Dobbe 2023), a reliance on predefined ethical principles applied reactively and at the level of individual responsibility (Young 2003; 2011; Robaey 2013; 2016), and often reproducing anthropocentric framings of moral concern (Young 2000). Although the reviewed scholarship predominantly condemns AI’s negative environmental impact, there is little explicit reflection on the value of sustainability itself, and ethical concerns and ways forward remain human-centered.

An interdisciplinary body of scholarship increasingly argues that more-than-human approaches are needed to address the ethical challenges posed by sociotechnical systems such as AI (Cila et al. 2017; Coeckelbergh 2012; Nyholm 2020; Overbeeke et al. 2003; Rijssenbeek 2025). By foregrounding the interdependence of humans, non-human animals, material infrastructures, and ecological environments, these approaches expand ethical reflection and intervention beyond anthropocentric frameworks, a crucial perspective broadening given the urgency and complexity of AI’s environmental impact.

More-than-human perspectives on AI are beginning to emerge across design, ethics, and human–computer interaction (e.g., Giaccardi and Redström 2020; Stead et al. 2022; Kudina 2024; Nicenboim et al. 2025). While diverse, they often converge on relational and care-based logics that emphasize situated and interdependent human-world relations (Gilligan 1982; Tronto 2020), increasingly extended to technological and ecological domains (de la Bellacasa 2017; Van Wynsberghe 2020; Ley 2023; Kudina et al. 2025). In these frameworks, ethics emerges dynamically within relational entanglements rather than from external predetermined principles, allowing for anticipatory and structural forms of moral responsibility.

Adopting more-than-human logic resonates with several omissions identified in our scoping review, including limited attention to water use in AI development and insufficient interdisciplinary engagement. These insights are further underscored by growing public opposition to AI infrastructures and related political tensions (Hamilton 2022; Seesel 2023; Verma and Tan 2024; Fleury and Jimenez 2025). Our scoping review shows that there is flexibility and room to innovate in the ethics of Sustainable AI, complementing established principle-based and justice-oriented approaches with care-based, relational, intercultural, and environmental ethics. Doing so may not be straightforward but is morally desirable, given the global political will to increase the pace of AI development alongside worsening environmental degradation.

## Conclusions

Although most literature on Sustainable AI focuses on AI for sustainability, research on the sustainability of AI, and specifically on its environmental impact, has rapidly increased in the past five years. In this scoping review, we studied (1) how the environmental impact of AI is reported on and by which disciplinary domains; (2) who is ascribed responsibility for mitigating said impact; (3) what solutions are proposed; and (4) what implicit and explicit normative commitments are made by authors in these studies. The results allow for a nuanced analysis of where the field currently stands, and of gaps and opportunities for further research.

The results show that most research on the environmental impact of AI is conducted by computer scientists, engineers, natural and formal scientists, who view the environmental impact as a problematic resource-intensive property of AI technology. A wide array of the proposed solutions targets developers and programmers and, to a lesser extent, policymakers. Most contributions point towards (the need for) technological solutions, revealing a technofix attitude in the scholarship, followed by (the need for) tools and methods to measure transparency, more reflection and deliberation on the issue, and institutional rules and guidelines. Overall, most authors seem to suggest that, with the implementation of proposed solutions, AI can become more environmentally sustainable; very few authors question the desirability of large-scale AI systems in societies altogether. While there was an overall lack of explicit ethical reflections in the selected scholarship, nevertheless, many authors point towards ethical issues, questions, and dilemmas when considering the environmental impact of AI, e.g., injustices and trade-offs between environmental costs and benefits of AI. Few authors draw further on ethical concepts and theories, and if they do, there seems to be a bias towards anthropocentrism and a preference for principle-based ethics generally or focusing on justice specifically.

Our scoping review and the accompanying discussion allow us to propose several avenues for future research. *First*, our study revealed rampant conceptual ambiguity regarding both the term “AI” (e.g. solely technical or systematic sociotechnical understandings) and the term “sustainability,” which complicated work in studying the environmental impact of AI. We suggest paying attention to the language pragmatics in disciplinary contributions to gauge an overlap in meanings, and also looking towards international standards to ground interdisciplinary work in this field. *Second*, we identified an opportunity for the humanities, including AI ethics, philosophy, and the social sciences, to engage with the topic of the environmental sustainability of AI. Connectedly, reducing the environmental impact of AI can be considered a wicked problem, involving not only technological, but also economic, social, political, legal, and ethical aspects, and therefore, interdisciplinary endeavors are essential. This would imply going beyond a direction of technological solutions and calls for transparency to also engage with the systematic origins and effects of the environmental impact of AI. To make this achievable, *third*, we made a call towards collaborative research efforts under interdisciplinary consortia-type alignments to prevent disproportional burden on individual researchers. *Fourth*, and speaking to our main objective in this paper, we identified a need to ethically innovate in the space of sustainability of AI to mirror its fundamentally more-than-human and interdependent nature. We suggested exploring the possible contributions that relational, care, feminist, intercultural, and environmental ethics can make here, in view of their focus on ethics as emerging within complex interdependencies and recognizing ethical agency beyond humans.

Finally, *fifth*, our review points to a need to scrutinize fundamental ethical questions about further AI development. Is it at all necessary? In what cases is the environmental impact of AI justified? How to deal with the many ethical trade-offs and injustices already identified through the scholarship and in practice? We want to stress that the environmental impact of AI is only the tip of the iceberg, as the problem of mineral extraction, water, and energy use is a feature of cloud-based technologies in general, and thus intrinsically intertwined with the digitalization of societies. Therefore, tackling such fundamental ethical questions would require conversations across disciplines and a coordinated approach, but first of all, it would require daring to raise them. By sketching the contours, dominant framings, normative underbelly, gaps, and opportunities of the scholarship on the environmental impact of AI, this scoping review contributes to this project.

## Data Availability

No datasets were generated or analysed during the current study.

## Footnotes

1 Notably, PRISMA (Preferred Reporting Items of Systematic reviews and Meta-Analyses) as a protocol for systematic reviews, https://www.prisma-statement.org/protocols.

2 Skaug and Sellinger contrast the concept of a technofix with techno-solutionism, which is a technology-driven approach to solutionism, “characterized by confidence in human reason and our capacity to find and implement numerous strategies for overcoming obstacles and capitalizing on opportunities, typically through courses of action that significantly alter social norms” (Skaug and Sellinger 2024, p.12). In the context of this paper, the term “technofix” applies better than “techno-solutionism,” because the papers that propose technological solutions do not propose significant changes in social norms.
